# Avian influenza (H5N1) virus, epidemiology and its effects on backyard poultry in Indonesia: a review

**DOI:** 10.12688/f1000research.125878.2

**Published:** 2023-02-17

**Authors:** Saifur Rehman, Mustofa Helmi Effendi, Adiana Mutamsari Witaningruma, Ugbo Emmanuel Nnabuikeb, Muhammad Bilal, Asghar Abbas, Rao Zahid Abbas, Kashif Hussain

**Affiliations:** 1 Division of Veterinary Public Health Faculty of Veterinary Medicine, Universitas Airlangga, Surabaya, East Java, 60115, Indonesia; 2 Department of Applied Microbiology, Faculty of Science, Ebonyi State University, Abakaliki,, Nigeria; 3 Department of Epidemiology and Public Health, University of Veterinary and Animal Sciences, Lahore, Islamic, 40050, Pakistan; 4 Faculty of Veterinary Medical Sciences, University of Calgary, Alberta, Canada; 5 Department of Pathobiology, Muhammad Nawaz Sharif University of Agriculture, Multan, Islamic, Pakistan; 6 Department of Parasitology, University of Agriculture, Faisalabad, Islamic, Pakistan

**Keywords:** Avian influenza, backyard poultry, Public health, H5N1, Indonesia

## Abstract

Avian influenza (AI) is a zoonotic viral endemic disease that affects poultry, swine, and mammals, including humans. Highly pathogenic avian influenza (HPAI) is caused by influenza type A virus subtypes H5, and H7 which are naturally carried by a wild bird and often affect domestic poultry. Avian influenza (AI) is a major problem worldwide that causes significant economic losses in the poultry sector. Since 2003, the widespread H5N1 HPAI in poultry has led to high mortalities resulting in huge economic losses in the poultry sector in Indonesia. Domestic poultry is a key source of income that contributes to economic growth, both directly and indirectly, by reducing poverty among the people living in rural communities. Furthermore, in many developing countries, including Indonesia, rural people meet a portion of their food needs through backyard poultry. Nevertheless, this sector is strongly affected by biosecurity hazards, particularly in Indonesia by HPAI infections. Avian influenza (AI), subtype H5N1 has zoonotic significance, posing major risks to public health and poultry. Due to close interaction between wild migratory birds and ducks, the domestic poultry sector in Indonesia is directly affected by this virus. This virus continues to be ubiquitous in Indonesia as a result of the unpredictable mutations produced by antigenic drift and shift, which can persist from a few days to several years. In this review, the epidemiology and impact, of highly pathogenic avian influenza H5N1 subtype virus infection on backyard poultry in Indonesia were discussed.

## Introduction

Highly pathogenic avian influenza (HPAI) virus subtype H5N1 has caused multiple outbreaks in poultry worldwide and hundreds of (mostly fatal) human cases since its discovery in Hong Kong in the late 1990s. ^1^ ^–^ ^4^ Avian influenza virus (AIV) affects a variety of animals, including birds, horses, dogs, cats, whales, and pigs, and have zoonotic potential that causes death in humans. Avian influenza A virus subtypes (including H5N1 and H9N2) have caused significant economic losses in the poultry sector, especially in the backyard and commercial poultry farming around the world. Massive vaccinations have been used to reduce the number of avian influenza infections, but, no appropriate precautionary measures were adopted in the domestic poultry industry. Highly pathogenic avian influenza (HPAI) strain H5N1 has been found in domestic poultry or wild birds from 61 countries since the isolation of HPAIV subtype H5N1 from a domestic goose in Guangdong Province, China (A/goose/Guangdong/1/96). ^5^ HPAI outbreaks were reported in seven of the eleven Southeast Asian countries between 2003 and 2008. In December 2003, Vietnam was the first country to report poultry death caused by H5N1 HPAIV infection. ^6^ Although the disease was first reported in Indonesia in January 2004, a retrospective investigation suggests that outbreaks in backyard poultry in Indonesia began in August 2003. ^7^ Similarly, the infection could have existed in Vietnam before December 2003. In several Southeast Asian countries, high poultry mortalities due to other illnesses, such as Newcastle disease, are widespread, which may have contributed to HPAI detection and diagnosis delays. ^6^ Domestic bird losses in the area were estimated to be 140 million in 2005, at a cost of almost US$10 billion. ^8^ The backyard and commercial poultry farming play an important role in many Asian countries in providing a suitable percentage of protein in the form of meat and eggs. The increasing population will immediately increase the food requirement percentage items all over the world. As a result, this source has evolved into a strong source of energy between the supply and demand for animal protein. Rural poultry has made a substantial contribution to poverty alleviation in many industrialized and developing countries. ^9^ The highly pathogenic avian influenza virus subtype H5N1 affects all segments of the chicken population, including commercial and domestic poultry, and the risk of infection spread increases with wild migratory birds, which have no restrictions on crossing international boundaries. ^9^ However, farmers’ ignorance and lack of knowledge of the systematic source of this virus play a key part in the spread of infection. ^10^ ^,^ ^11^ Women play a key role in the development of the backyard poultry production system in many regions of the world, allowing them to meet their economic demands regularly by growing birds at home. ^12^ HPAI H5N1 has been endemic in Indonesian poultry since 2003, resulting in severe economic losses for both the poultry industry and backyard farms. In high-incidence areas, the disease has been detected in 32/34 provinces, ^13^ ^,^ ^14^ resulting in the deaths of millions of birds and the closure of numerous farms. ^15^ According to the universal naming scheme for the HA gene of the HPAI H5N1 virus, the hemagglutinin (HA) genes evolved from clade 2.1 into several subclades while HPAI H5N1 viruses were continually distributed among poultry in Indonesia from 2003 to 2010. ^16^ Vaccination programs have been used to restrict the spread of HPAI H5N1, however, due to low vaccination coverage and the use of unlicensed vaccines, they have not proven successful. ^17^

In this review, epidemiology and impact of highly pathogenic avian influenza H5N1 subtype virus infection on backyard poultry in Indonesia were discussed. The epidemiology, evolutionary history, detection method, and threat level of avian influenza A virus subtype H5N1 in backyard poultry of Indonesia are discussed in detail. Future initiatives and policies to reduce the risk of avian influenza (HPAI and LPAI) in Indonesian backyard poultry are suggested.

## Epidemiology and life cycle

Avian influenza is a contagious viral infection that affects poultry, animals, and humans worldwide. The majority of human infections were caused by type A and B influenza viruses, while poultry was only infected by type A influenza. A number of strains of the avian influenza virus (LPAI and HPAI) have been detected in poultry farms around the world. ^18^ In 1996, the H5N1 virus, which is a type of HPAI, was found in geese in China. In 1997, during a poultry outbreak in Hong Kong, Asian H5N1 was discovered in humans for the first time. Since then, it has been found in humans, poultry, and wild birds in over 50 countries throughout Africa, Asia, Europe, and the Middle East. ^19^ ^–^ ^22^ The presence of living cells is necessary for the spread of viruses.

The influenza virus has a multistep process for reproduction and infection. PB2 plays a role in RNA replication via identification of the mRNA cap, PB1 helps in RNA extension throughout replication. PA is component of polymerase that help in endonuclease function. Haemagglutinin (HA or H) plays a role in host cell virus attachment and subsequent fusion with cell membranes, while neuraminidase (NA or N) promotes the release of viruses from the surface of the host cell by hydrolyzing glycoprotein sialic acid, which helps to release particles of the progeny virus particles from host cells. ^23^ NP is the protein that binds to RNA; nuclear import control. Non-structural protein 1 (NS1) plays a major role in inhibiting host immune response through interferon (IFN) production limitation. ^24^ NS2 referred to as a nuclear export protein or NEP plays a role in the export of RNPs during viral replication from the nucleus to the cytoplasm, and also regulates the transcription and replication processes of viruses. ^25^ The dominant structural protein is matrix protein 1 (M1), the major structural protein that also plays an important role in the assembly and budding of viruses in determining virus morphology. ^26^ Matrix protein 2 (M2) is the pH-regulating ion channel and is responsible for virus uncoating, the phase following the entry of the virus into the host cell. ^27^ In addition, in the last stage of the viral life cycle, this protein also plays an important role in membrane splitting. ^28^ Matrix protein 42 (M42) can replace M2 functionally and promote effective replication in null M2 influenza viruses. ^29^ The incubation period of the disease in chickens is one to seven (1-7) days. The most prevalent sialic acid links with which influenza viruses have a strong affinity are 2,3 and 2,6 linkages. One aspect of host specificity could be the various sialic acid connections. Both types of receptors are widely expressed in chickens, ducks, cats, and pigs, with SA 2,6Gal being the most abundant in human respiratory tissues, including epithelial cells in the nasal mucosa, paranasal sinuses, pharynx, trachea, bronchi, and bronchioles ^30^ ^–^ ^33^ while SA 2,3Gal is occasionally found in the nasal mucosa and non-ciliated cuboidal bronchiolar cells at the junction of the respiratory bronchiole and alveolus, SA 2,3Gal is rarely found in the pulmonary bronchiole and alveolus. ^34^

## Virus entry and release

The virus attaches to its host cell for the first time via N-acetyl neuraminic (sialic) acid, a nine-carbon acidic monosaccharide. ^35^ The most common sialic acid linkages with which influenza viruses have a strong affinity are 2,3 and 2,6 linkages. ^33^

**Figure 1.**
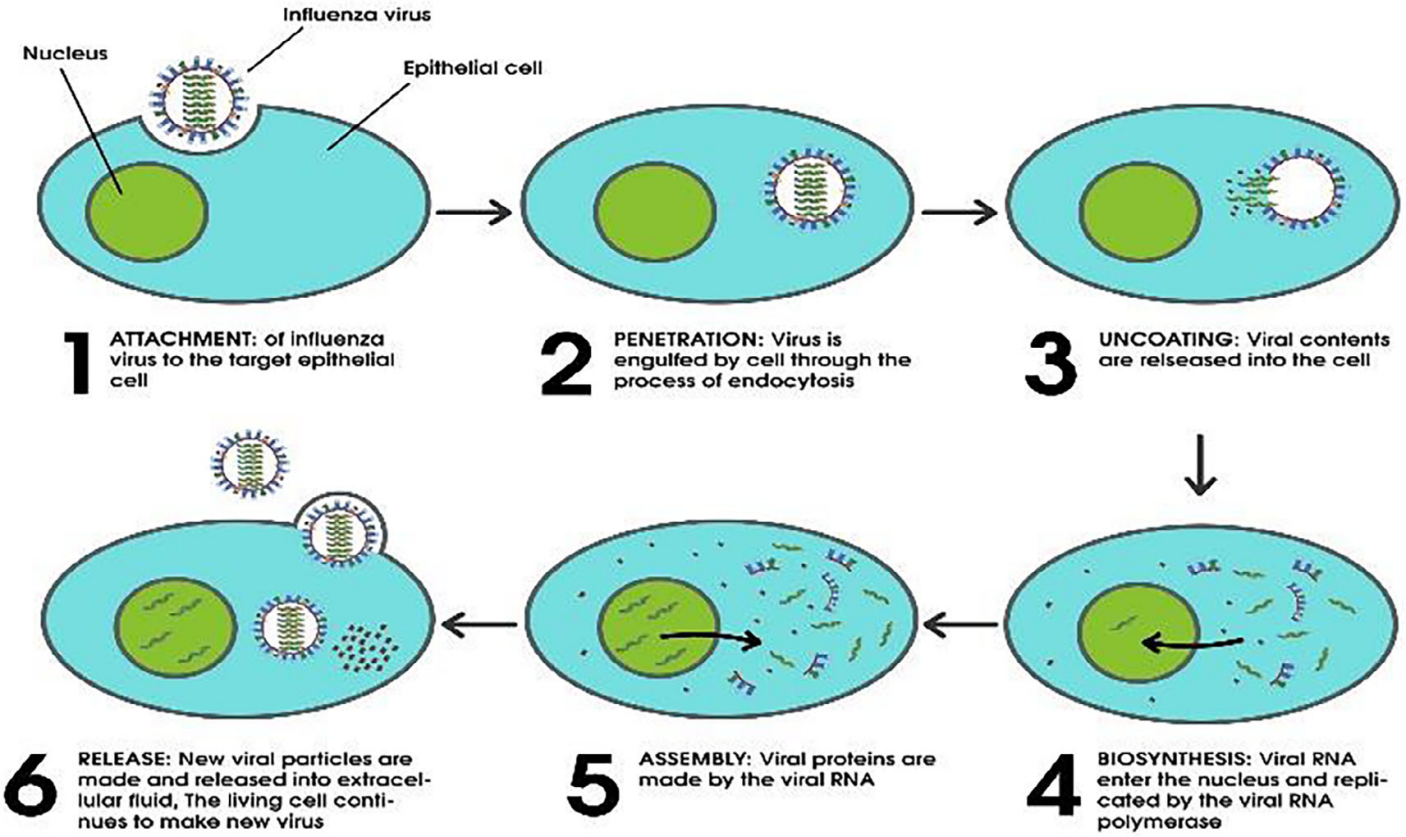
The life cycle of avian influenza A virus has been reproduced from the viral life cycle. ^54^

When an influenza virus infects a host cell, it produces the HA glycoprotein as a precursor, HA0, that’s cleaved into subunits (HA1 and HA2) by using host serine proteases before virus particles become infectious. ^36^ The virus enters the host cell through a process called receptor-mediated endocytosis, which takes place at the inner face of the plasma membrane and results in the formation of an endosome. ^37^ ^,^ ^38^ This approach not only induces the HA0 conformational change but also opens the M2 ion channel during the fusing of viral and endosomal membranes. This allows the virion interior to become acidic, which frees the vRNP from M1 when it is occurring during the fusion process. This makes it possible for the vRNP to gain access to the cytoplasm of the host cell. ^39^ ^,^ ^40^ Through a process referred to as “cap snatching,” the mRNA is able to obtain a 5’ capped primer. The PB2 protein performs a crucial role in grabbing this primer from the host mRNA. ^41^ To begin viral translation by ribosomes, the resulting positive sense viral mRNA is transferred to the cytoplasm through nuclear pores. ^42^ The cytoplasm of the host cell produces polymerase basic (PB1 and PB2), nonstructural (NS1 and NS2), NP, PA, and M1 proteins, which are then transported to the nucleus to take part in matrix and nonstructural splicing, transcription, and replication. Ribosomes are responsible for the synthesis of surface glycoproteins (HA and NA), which are subsequently transported to the endoplasmic reticulum (ER), where they undergo glycosylation before being pleated in the Golgi apparatus. ^43^ In the nucleus of the infected host cell, freshly generated PB1, PB2, PA, NP, and NS2 proteins combine to form new vRNP complexes. After establishing M1-vRNP complexes, M1 proteins accelerate the transport of vRNP to the cytoplasm. The nuclear export signal (NES), which is carried by NP proteins and blocked by M1 proteins, directs the nuclear export of vRNA complexes. As a result, newly created vRNA and M1 proteins are unable to enter the nucleus once more. ^44^ The process of budding and release complete viral replication. The process of budding takes place at the apical plasma membrane of the host cell, and it is thought that this process is likely kicked off by the buildup of M1 peptide on the cytoplasmic side of the lipid bilayer. The protein complexes that are represented by M1 can communicate with the cytoplasmic terminals of envelope proteins (M2, HA, and NA proteins). The creation of a bud and an assembly site in the cellular membrane is brought about as a result of this interaction. ^45^ The most important process that occurs before the nascent virion leaves the plasma membrane is the cleavage of sialic acid residues from glycoproteins and glycolipids by NA. This process speeds up how quickly virus particles are released into extracellular medium ( Figure 1). ^46^

Initial surveillance of avian influenza viruses in Indonesia found a significantly higher prevalence of HPAI H5N1 at live bird markets (LBMs) than in poultry-producing areas, implying that the HPAI virus must move widely during the trade process. ^47^ ^,^ ^48^ Moreover, the value chain of backyard poultry (e.g., Kampung or indigenous chickens) and commercial poultry (e.g., broilers and layers) marketed in Indonesian urban live bird markets (LBMs) differs significantly. Backyard poultry is typically bought by middle-class or small-scale poultry dealers who go by motorbike to several villages to trade with farmers or purchase birds from small village markets (Food and Agriculture Organization, unpublished data). To provide greater insight into the epidemiology of HPAI in Indonesia, genetic and antigenic data are significant. Epidemiological research on duck scavenging in smallholder farms in central Java, Indonesia, highlighted that these birds could be an important source of the H5 virus for indigenous chickens. ^49^ In 2004 there were 7 pandemics in Indonesia involving 4 types of the H5N1 virus, but nothing was seen to be transmitted to the Sumatera region. ^50^ In 2004, the phylogenetic analysis of the H5N1 pandemic in Bangka Belitung was genotypically different from the other 6 regions outside Sumatra. Meanwhile, two new H5N1 virus clades were introduced in Sumatra in 2005 and distributed through Riau, Jambi, Palembang, and Lampung in 2005. ^51^ Clade 2.1 viruses have been enzootic in Indonesia since 2003. However, during poultry outbreaks since 2012, a new HPAI H5N1 clade 2.3 virus has been found. To date, a new H5N1 subclade (2.3.2.1) has evolved, and a novel vaccine based on isolate A/duck/Sukoharjo/BBVW-1428-9/2012 has been produced. ^52^

The mutation was very similar to the variant of H5N1 found at almost the same time in West and East Java, Bali, and Nusa Tenggara Barat (NTB). The possibility of transmission between these areas is therefore very high. ^51^ In 2004, FAO identified four poultry production sectors globally: sector 1: industrial and integrated production, sectors 2 and 3: other commercial production systems, village or backyard production with birds or products consumed locally include in sector four. ^53^ The first phase of the participatory disease surveillance response (PDSR) project from January 2006 to April 2008 emphasized the detection and control of HPAI by separate participatory disease surveillance (PDS) and participatory disease response (PDR) teams primarily in sector 4 poultry at the household level. Through the participatory disease surveillance response (PDSR) program, outbreak control, and prevention capacity in village-based poultry have been developed across endemic areas of Indonesia. ^53^

**Figure 2.**
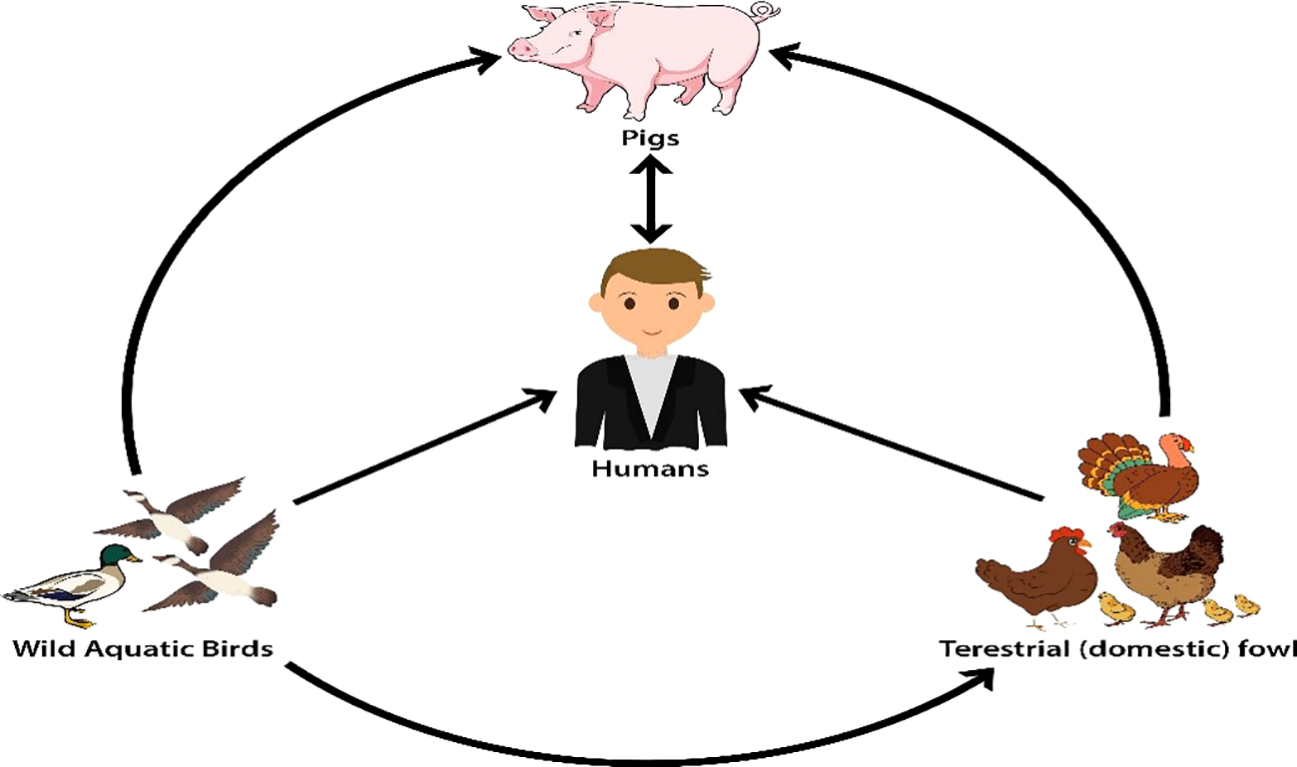
The reservoir of influenza A viruses has been reproduced with permission from Al-Mubarak *et al.*, 2014. ^41^

^41^

All influenza A subtypes are naturally found in wild aquatic birds. Viruses of avian influenza A are often transmitted from wild birds to domestic poultry and from domestic poultry to pigs. The influenza A virus can reassort in pigs from avian, swine, and human sources, and pigs are frequently exposed to human and domestic poultry virus strains. Humans might be affected by influenza A viruses from pigs act as mixing vessels for the transmission of these viruses ( Figure 2).

## Virus identification

There are numerous diagnostic techniques available for detecting avian influenza viruses in respiratory tissues, and notably molecular assays (PCR). Haemagglutination inhibition and ELISA are serological tests that are frequently used to identify antibodies of influenza A and B viruses. As a consequence, proper influenza serological testing involves the collection of matched acute and convalescent samples 2-3 weeks apart in order to identify a 2 or 4-fold or more elevation in influenza virus strain-specific antibodies. ^55^ Rapid influenza diagnostic tests (RIDTs) are immunoassays that can detect influenza A and B viral nucleoprotein antigens in respiratory samples and display the results in a qualitative manner. ^56^

## Effects of HPAI A virus subtype H5N1 on backyard poultry

Backyard poultry farming is a conventional method of maintaining chickens that are mostly used in rural areas. It is a low-input enterprise that involves raising small flocks of poultry birds in backyards using a free-range system in which the birds forage for food. ^57^ In developing countries, small-scale poultry is raised by family members utilizing available locally mixed feed resources. Backyard chickens typically wander around more inside and outside the house, scavenge for food, and share it with other wild birds. ^58^ Mostly every rural and urban family owns 5-20 adult chickens in a small flock, which are primarily cared for by children and women. Profits are often modest, and products are consumed for their use presented as religious offerings, or given as gifts. ^59^ ^,^ ^60^ In most Asian countries, AI has an impact on all aspects of the poultry industry, but it appears to be most widespread in industrial ducks, rural chickens, live bird markets, and fighting cocks. ^61^ The majority of infections spread across backyards and other commercial and wild migratory birds contribute to the global spread of the high pathogenic subtype H5N1 virus. Wild migratory birds act as a reservoir host for AI viruses that become a formidable source of AIV infection all over the world. Indonesia’s poultry-producing industry is extremely diversified. Village or backyard production with birds or products consumed locally include in sector four. ^53^ AIV subtype H5N1 was introduced to Indonesia on multiple occasions. ^62^ The virus was likely propagated by wild migratory birds, as well as through trade and transportation of poultry and poultry products between different places. ^63^ ^,^ ^64^ A higher level of backyard poultry contact is likely linked to the nature and purpose of visits, which disclose additional farm-to-farm and farm-to-live bird market tours aimed at observing birds or acquiring live birds and items. This shows that Indonesia’s Sector 4-(backyard farms) has the highest risk of being infected with HPAIV from other poultry sectors and of being a possible infection source, particularly for the small-scale commercial poultry farms. ^65^ ^,^ ^66^

In Indonesia, Sector 4-(backyard farms) was shown to have a higher incidence of HPAIV infection and a higher proportion of disease outbreaks, suggesting that they may play a role in maintaining the HPAIV infection cycle in poultry. ^65^ ^,^ ^67^ ^,^ ^68^ In most years, HPAI H5N1 poultry epidemics in backyard poultry in Indonesia peak in January or February(USAID Indonesia, unpublished data). Backyard poultry production accounts for around 50% of Indonesia’s total chicken population. ^69^ Backyard chicken raising in Indonesia is connected with poor sanitation and biosecurity, which appears to pose a considerably higher risk of HPAI virus transmission. ^70^ According to Loth *et al.*, participatory disease surveillance (PDS) study in backyard chickens found that “human population density” and “rice cultivation” had a significant association with HPAI cases in Indonesia. Reassortment of HPAIV has largely occurred in backyard chickens in Indonesia. Reassortment may have occurred in West Java due to high poultry density, the existence of many poultry kinds, and frequent contact between poultry farms and domestic poultry and wild birds. ^71^ Domestic poultry is described as free-ranging birds living in a limited space alongside humans and other animals. These backyard birds can also scavenge other types of food from wild migrating birds, which can spread HPAI infection. ^72^ In both developed and developing countries, this industry is one of the most important contributions to poverty alleviation. Children and women raise poultry in a relatively constrained area of the home due to the cheap adjustable cost and rapid changeover of productive output. In general, backyard poultry serves as a source of income and savings in Indonesia, where chickens/ducks can be sold to pay for children’s school fees and other household emergency needs such as medical care. ^73^ Around 300 million chickens, ducks, and quails are believed to be kept in the backyards of 30 million Indonesian homes, or 60% of the country’s total population. ^74^

According to participatory disease surveillance response (PDRS) findings, the village attack rate in Bali (number of villages reporting H5N1 in S4 poultry/total number of villages) was 12.74% (92/722) in 2009 and 28.26% (204/722) in 2007 (PDSR, unpublished data, 2010). To estimate the frequency of H5N1 in backyard poultry, a survey was done in Bali. 14 of 1714 collected faecal samples from afflicted villages were positive for H5N1 after viral isolation. ^75^ Due to higher H5N1 frequency in these places than in Bali, inferring surveillance data for semi-intensive poultry farms in other Indonesian provinces, such as Java, is particularly difficult. ^76^ ^,^ ^77^ Desniwaty Karo-karo *et al.* conducted a study among different bird species to determine the prevalence of HPAI H5N1 in the Indramayu and Subang regencies of West Java Province. The findings of their research stated that the biggest peak AI occurred in February 2016 (average 41.3%, 95% confidence interval: 25.6–56.5%), with the majority of positive samples coming from backyard poultry (average 69.23%, 95% confidence interval: 54.74–83.71%). ^14^ E. Basuno *et al.* reported that smallholders and backyard farmers in Indonesia suffered enormous financial losses as a result of the HPAI outbreak. These losses were due to high mortality, decreased production, less demand for poultry products, and a decrease in price specifically in backyard poultry. ^15^

Another study conducted in West Java among backyard chickens’ ducks and other birds observed backyard chickens have (average 59%, 95% confidence interval: 49–69%) with the highest death rate, followed by ducks (average 32%, 95% confidence interval: 19–45%) and others (average 28%, 95% confidence interval: 16–40%). Backyard poultry had the highest observed morbidity, at 44% (95% confidence interval: 32–54%), whereas ducks had 28% (95% confidence interval: 17–38%). ^78^ Although the role of wild bird migrations in the spread of H5N1 across the Indonesian archipelago cannot be ruled out, migrating ducks may have carried the virus to Java. Agricultural techniques and the chicken trade, on the other hand, are believed to have kept the virus alive in Indonesia. ^79^ In Bangladesh, the seroprevalence of AI virus was reported to be 20% in chickens and 23% in flocks, whereas in Vietnam, the seroprevalence of H5 was found to be 17.5% at the bird level in backyard poultry and smallholder commercial duck farms. ^80^ ^,^ ^81^ Avian influenza was also reported in backyard poultry to be (1.5% out of 100 birds in Thailand ^82^ and a high prevalence (62.5%) in Pakistan. ^83^ As a result of the free-range nature of these birds, backyard poultry is primary source of AI infection in this poultry farming system. Food is readily available, which attracts wild migrating birds to backyard poultry enterprises.

## Conclusion

The epidemiology, molecular mechanisms used by highly pathogenic avian influenza H5N1 to cause pathogenicity, and risk scale of avian influenza in Indonesian backyard poultry were reviewed. Research articles reviewed from different areas of Indonesia indicated that H5N1 infection is endemic in backyard poultry. It was observed that backyard poultry is an important source of transmission of H5N1 disease to other sectors of poultry farms in Indonesia because of the poor sanitation, lack of biosecurity system, and easy mixing of free-range birds with the wild long-distance migrating birds. The primary goal of this review is to determine the impact of highly pathogenic avian influenza subtype H5N1 in backyard poultry in Indonesia, as well as identify high-risk locations or villages for ongoing monitoring and effective control of AI viruses in hotspot areas, as well as define the backyard production system. The existence of the HPAI virus subtype H5N1 in many parts of Indonesia revealed the AIV exposure among backyard chickens. A broad vaccination strategy at the route level is required for the control of H5N1 infection in backyard chickens to prevent the early phase of infection and limit the danger of avian influenza transmission. Regular surveillance of backyard poultry is required because these birds are at a higher risk of contracting the infection. Washing hands after handling birds, especially backyard and other fancy birds, is vital for human infection control, and this will likely reduce the occurrence of infection among them. Mass surveillance will help determine the optimal time for avian influenza viruses to infect backyard poultry and lower the percentage of cases.

## Data availability

No data are associated with this article.
