# Supplementary material for: Avian influenza (H5N1) virus, epidemiology and its effects on backyard poultry in Indonesia: a review
Source: F1000Res. 2023 Feb 17;11:1321. Originally published 2022 Nov 15. [Version 2] doi: 10.12688/f1000research.125878.2 (PMC9947427; doi:10.12688/f1000research.125878.2)
Supplement: Peer review history

# Peer review: Reviewer response for version 2

**DOI:** 10.5256/f1000research.143671.r163876

**Published:** 2023-02-22

**Authors:** Gangil Rakhi

**Affiliations:** 1 Department of Veterinary Microbiology, College of Veterinary Science and Animal Husbandry MHOW, NDVSU, Jabalpur, Madhya Pradesh, India

This article is corrected now. There is no further correction required. In my opinion it can be approved for indexing.

Is the review written in accessible language?

Partly

Are all factual statements correct and adequately supported by citations?

Yes

Are the conclusions drawn appropriate in the context of the current research literature?

Yes

Is the topic of the review discussed comprehensively in the context of the current literature?

Yes

Reviewer Expertise:

Virus isolation and Development of diagnostics for identification of virus, Veterinary Microbiology

I confirm that I have read this submission and believe that I have an appropriate level of expertise to confirm that it is of an acceptable scientific standard.

# Peer review: Reviewer response for version 2

**DOI:** 10.5256/f1000research.143671.r163875

**Published:** 2023-02-20

**Authors:** Sahibzada Waheed Abdullah

**Affiliations:** 1 State Key Laboratory of Veterinary Etiological Biology, National Foot-and-Mouth Disease Reference Laboratory, Lanzhou Veterinary Research Institute, Chinese Academy of Agricultural Sciences, Lanzhou, China

The authors have responded to all the comments. I suggest accepting the manuscript in its current form.

Is the review written in accessible language?

Partly

Are all factual statements correct and adequately supported by citations?

Partly

Are the conclusions drawn appropriate in the context of the current research literature?

Partly

Is the topic of the review discussed comprehensively in the context of the current literature?

Yes

Reviewer Expertise:

NA

I confirm that I have read this submission and believe that I have an appropriate level of expertise to confirm that it is of an acceptable scientific standard.

# Peer review: Reviewer response for version 1

**DOI:** 10.5256/f1000research.138231.r159652

**Published:** 2023-01-26

**Authors:** Gangil Rakhi

**Affiliations:** 1 Department of Veterinary Microbiology, College of Veterinary Science and Animal Husbandry MHOW, NDVSU, Jabalpur, Madhya Pradesh, India

The review on "Avian influenza virus, epidemiology and its effect on backyard poultry in Indonesia: a review" is interesting and gives much more information about status of Avian influenza outbreaks in Indonesia. This review can be considered for indexing with some corrections. 

1. Line no. 4: **Avian influenza type A **virus should be written instead of AI A virus (because first time strain should be clearly mentioned)
2. Second paragraph of Introduction:   Line 2...it should be **were** discussed instead of was discussed. Line 3....it should be **are **discussed in details instead of is discussed in detail. rewrite the last sentence of this paragraph (Recommendations for future.........backyard poultry).
3. Second paragraph of Epidemiology and life cycle: full form of LBM should be write first time then abbreviation can be used. 
  In this paragraph **phylogenetic analysis and different clades of AIV strains **were discussed. So it is suggested that **phylogenetic tree should be included in this paper **which is discussed.
  In this paragraph according to author **FAO identified four poultry sectors globally whereas other three sectors are not discussed.**
**PDSR describe first**
  T **he last paragraph of Epidemiology and lifecycle should comes first.**
4. First paragraph Viral identification: this paragraph included (clad 2.1 virus....has been produced) discussion about clad of phylogenetic tree. **So this paragraph should be included in above paragraph of epidemiology where phylogenetic analysis discussed**. 

Is the review written in accessible language?

Partly

Are all factual statements correct and adequately supported by citations?

Yes

Are the conclusions drawn appropriate in the context of the current research literature?

Yes

Is the topic of the review discussed comprehensively in the context of the current literature?

Yes

Reviewer Expertise:

Virus isolation and Development of diagnostics for identification of virus

I confirm that I have read this submission and believe that I have an appropriate level of expertise to confirm that it is of an acceptable scientific standard, however I have significant reservations, as outlined above.

# Response

**Published:** 2023-01-27

**Authors:** Saifur Rehman

**Affiliations:** UNAIR, Indonesia

Dear Reviewer, 

 Thanks for your valuable comments and suggestions on the manuscript entitled:

**“Avian influenza virus, epidemiology and its effect on backyard poultry in Indonesia: a review**

 Line no. 4: **Avian influenza type A **virus should be written instead of AI A virus (because first-time strain should be clearly mentioned)

**Response: **We have corrected by following your valuable suggestions

 Second paragraph of Introduction:   Line 2...it should be **were** discussed instead of was discussed. Line 3....it should be **are **discussed in details instead of is discussed in detail. rewrite the last sentence of this paragraph (Recommendations for future.........backyard poultry).

**Response**: We have corrected these mistakes in the mentioned paragraphs

 Second paragraph of Epidemiology and life cycle: full form of LBM should be written first time then abbreviation can be used. 

**Response: **We have mentioned full form of LBM

 In this paragraph **phylogenetic analysis and different clades of AIV strains **were discussed. So it is suggested that **phylogenetic tree should be included in this paper **which is discussed.

**Response:** Dear reviewer thanks for your valuable suggestions we have not found any phylogenetic tree like that we have found only paragraph which we have mentioned already in manuscript

 In this paragraph according to author **FAO identified four poultry sectors globally whereas other three sectors are not discussed.**

**Response: **We have mentioned all the four sectors

 PDSR describe first

**Response**: We have corrected accordingly

 The last paragraph of Epidemiology and lifecycle should comes first.

**Response:** We have corrected this paragraph by following both reviewer suggestions

 First paragraph Viral identification: this paragraph included (clad 2.1 virus....has been produced) discussion about clad of phylogenetic tree. **So this paragraph should be included in above paragraph of epidemiology where phylogenetic analysis discussed**. 

**Response:** We have moved this paragraph accordingly

# Peer review: Reviewer response for version 1

**DOI:** 10.5256/f1000research.138231.r155892

**Published:** 2022-11-21

**Authors:** Sahibzada Waheed Abdullah

**Affiliations:** 1 State Key Laboratory of Veterinary Etiological Biology, National Foot-and-Mouth Disease Reference Laboratory, Lanzhou Veterinary Research Institute, Chinese Academy of Agricultural Sciences, Lanzhou, China

An interesting review by Rehman et al. However, there are some issues the authors need to address before it could be considered for publication.

- Section introduction lines 1 and 2, Has instead of Had, its instead of their
- Line 6, Remove “Still,”
- Please modify the following sentence, Recommendations for future initiatives and policies to reduce the risk of avian influenza (HPAI and LPAI) in Indonesian backyard poultry.
- Please define LBMs at the first mention.
- The mutation was very similar to the variant of H5N1 found at almost the same time in West and East Java, Bali, and Nusa Tenggara Barat (NTB). Instead of “They have a mutation that is very similar to the variant of H5N1 found at almost the same time in West and East Java, Bali, and Nusa Tenggara Barat (NTB).”
- In the epidemiology and life cycle section, in 2nd paragraph, the authors mentioned, “FAO identified four poultry production sectors globally,” but the authors failed to describe the other three poultry production sectors.
- Define DPSR at first mention in the text.
- Define PDS at first mention in the text.
- The authors said that the Influenza virus has a multistep process for reproduction. I suggest that the authors first define all the viral proteins and then describe their roles in the virus life cycle. In fact, the virus life cycle paragraph is a disaster by mixing various viral proteins involved in the replication cycle. Authors are suggested to write this paragraph in an order from virus entry to release.
- The last paragraph of the epidemiology and life cycle should be moved to the start of this section.
- Please revise figure 1 by making some changes as follows; authors mentioned uncoating, but it was not shown in the figure. The receptor was still attached to the virus after the entry into the cytoplasm. The transcription stage occurs in the nucleus, but it was not shown, and the packaging step was also not shown. Provide a little detail of the figure in legend.

Is the review written in accessible language?

Partly

Are all factual statements correct and adequately supported by citations?

Partly

Are the conclusions drawn appropriate in the context of the current research literature?

Partly

Is the topic of the review discussed comprehensively in the context of the current literature?

Yes

Reviewer Expertise:

Virus-host interaction

I confirm that I have read this submission and believe that I have an appropriate level of expertise to confirm that it is of an acceptable scientific standard, however I have significant reservations, as outlined above.

# Response

**Published:** 2023-01-27

**Authors:** Saifur Rehman

**Affiliations:** UNAIR, Indonesia

Dear Reviewer, 

 Thanks for your valuable comments and suggestions on the manuscript entitled:

**“Avian influenza virus, epidemiology and its effect on backyard poultry in Indonesia: a review”**

 We welcome feedback. We have made modifications to the study on the following points:

 Section introduction lines 1 and 2, Has instead of Had, its instead of their            

**Response:** We have replaced had with has accordingly

 Line 6, Remove “Still            

**Response:** We have removed

 Please modify the following sentence, Recommendations for future initiatives and policies to reduce the risk of avian influenza (HPAI and LPAI) in Indonesian backyard poultry.

**Response: **We have revised this sentence accordingly

 Please define LBMs at the first mention

**Response:** We have mentioned the full name of LBMS

 The mutation was very similar to the variant of H5N1 found at almost the same time in West and East Java, Bali, and Nusa Tenggara Barat (NTB). Instead of “They have a mutation that is very similar to the variant of H5N1 found at almost the same time in West and East Java, Bali, and Nusa Tenggara Barat (NTB).”

**Response: **We have revised according to valuable suggestions

 In the epidemiology and life cycle section, in 2nd paragraph, the authors mentioned, “FAO identified four poultry production sectors globally,” but the authors failed to describe the other three poultry production sectors.

**Response: **We have mentioned all four sectors according to your suggestions

 Define DPSR at first mention in the text

**Response: **we have defined accordingly

 The authors said that the Influenza virus has a multistep process for reproduction. I suggest that the authors first define all the viral proteins and then describe their roles in the virus life cycle. In fact, the virus life cycle paragraph is a disaster by mixing various viral proteins involved in the replication cycle. Authors are suggested to write this paragraph in an order from virus entry to release.

**Response:  **All the viral proteins and then describe their roles in the virus life cycle according to your suggestions****

 The last paragraph of the epidemiology and life cycle should be moved to the start of this section.

**Response: **We have moved the last paragraph as you mentioned

 Please revise figure 1 by making some changes as follows; authors mentioned uncoating, but it was not shown in the figure. The receptor was still attached to the virus after the entry into the cytoplasm. The transcription stage occurs in the nucleus, but it was not shown, and the packaging step was also not shown. Provide a little detail of the figure in legend.

**Response: ** We have revised figure 1 by making new figures with your recommended changes
